# Adverse Events of DOACs in Children

**DOI:** 10.3389/fped.2022.932085

**Published:** 2022-07-04

**Authors:** Alessandra Bosch, Manuela Albisetti

**Affiliations:** ^1^Division of Hematology/Oncology, The Hospital for Sick Children, Toronto, ON, Canada; ^2^Division of Hematology, University Children's Hospital Zurich, University of Zurich (UZH), Zurich, Switzerland; ^3^Children's Research Center, University Children's Hospital Zurich, University of Zurich (UZH), Zurich, Switzerland

**Keywords:** adverse events, DOACs, children, pediatric, serious adverse drug events, bleeding complication

## Abstract

Venous thromboembolism (VTE) has an increasing rate of significance in pediatric patients. The currently standardized anticoagulants (unfractionated heparin, low molecular weight heparin and vitamin K antagonists) and their dose regimens were not comprehensively trialed in pediatric patients. Recently, several direct oral anticoagulants (DOACs) have been studied in clinical trials in the pediatric population and further trials are ongoing. Dabigatran etexilate and rivaroxaban results show that these DOACs are safe and efficient in the treatment and secondary prevention of pediatric VTE. This review will focus on adverse events (AEs) between specific DOACs reported in the clinical trials in children and compare them to standard of care. This will assist clinicians in decision making of selecting the right anticoagulation for their pediatric patients.

## Introduction

The incidence of venous thromboembolism (VTE) in pediatric patients has increased significantly in the past two decades. Improved clinical care of acutely ill children and neonates, as well as clinical awareness and refined diagnostic tools have led to an increase of VTE diagnoses in children (1).

The standard of care (SOC) for the treatment of VTE in pediatric patients to date has included unfractionated heparin (UFH), low molecular weight heparin (LMWH), fondaparinux, and vitamin K antagonists (VKA). These agents were not comprehensively studied in pediatric patients, and the treatment is mainly based on adult population studies, with dose regimens extrapolated from adult guidelines, as well as based on expert recommendations. This is not optimal, because the hemostatic system and the underlying medical conditions in children differ considerably from adults. These SOC anticoagulants are not convenient in their administration: UFH and LMWH require parenteral application (intravenous or subcutaneous), while VKA require dietary adjustments and constant laboratory monitoring (2, 3).

As a new form of therapy, direct oral anticoagulants (DOACs) have been studied over the last decade. As the name suggests, DOACs are administered orally, where no parenteral administration is required. A further benefit of these medications, is a fixed dosing regime, where no laboratory monitoring is necessary. Recently DOACs have been studied in phase 3 randomized clinical trials in the pediatric population and further trials are ongoing. Dabigatran etexilate and rivaroxaban results show that these DOACs are overall safe and efficient in the treatment of pediatric VTE, with similar recurrence risks and outcomes as the previous SOC, without an increased risk of bleeding. Studies in thromboprophylaxis for pediatric VTE and for children post-Fontan procedure showed similar outcomes for DOACs as compared to SOC. These trials are the largest studies of anticoagulant treatment in pediatrics to date and provide the basis for a new approach in the treatment of VTE in children (4–7).

This review will summarize and compare adverse events (AEs) between specific DOACs and SOC in children reported in Phase III trials. This will assist clinicians in decision making of selecting the right anticoagulation for their pediatric patients.

## Adverse Events (AE)

In the studies under consideration, AEs were defined as unexpected medical events that happened during treatment with the study drug. Serious AEs (SAEs) were defined as events related or leading to death, imminently life-threatening, persistent or significant disability or incapacity, or prolonged hospitalization. Bleeding events and deaths were reported separately from AEs/SAEs since these were considered outcome measures in the trials. AEs may not have been related or caused by the drug that was given, and may have been related to pre-existing conditions. AEs were recorded as percentages of participants experiencing AEs; some experienced more than one AE. Table 1 compares AEs and bleeding events of DOACs and SOCs in the considered pediatric phase III trials.

**Table 1:** Comparison of adverse events and bleeding events in pediatric DOAC trials.

**Study**	**DIVERSITY**		**Secondary Prevention**	**EINSTEIN JR**		**UNIVERSE**	
**Medication**	**Dabigatran**	**SOC**	**Dabigatran**	**Rivaroxaban**	**SOC**	**Rivaroxaban**	**ASA**
Nr of participants	176	90	203	329	162	76	34
Total adverse events (AEs)	77%	67%	75%	83%	77%	87%	85%
Death	0%	2%	0%	1%	0%	0%	0%
AE leading to treatment discontinuation	7%	3%	6%	1%	0%	4%	9%
Serious adverse events (SAE)	13%	20%	12%	*	*	32%	24%
Gastrointestinal AE	25%	4%	37%	33%	28%	33%	26%
Abdominal pain	7%	2%	10%	6%	6%	*	*
Vomiting	8%	1%	7%	11%	8%	*	*
Dyspepsia	6%	0%	6%	*	*	*	*
Pyrexia	6%	3%	6%	11%	8%	*	*
Headache	10%	4%	16%	17%	15%	*	*
Nasopharyngitis	6%	8%	17%	8%	5%	*	*
Infections	*	*	*	35%	30%	63%	65%
Total bleeding events	22%	24%	20%	*	*	36%	41%
Major bleeding events	2%	2%	2%	0%	1%	1%	0%
CRNM bleeding	1%	1%	1%	3%	1%	7%	9%
Minor bleeding events	19%	23%	18%	*	*	32%	35%
Gastrointestinal bleeding	5%	0%	2%	1%	1%	1%	3%
Epistaxis	5%	7%	6%	12%	11%	9%	9%
Gingival bleeding	1%	1%	1%	4%	1%	5%	3%
Bruising/skin bleeding	3%	7%	8%	*	*	30%	30%
Menorrhagia/vaginal bleeding	*	*	*	8%	4%	*	*

% of participants of the trial who experienced specific AEs or bleeding events. ^*^not reported /investigated in trial. SOC, Standard of Care; ASA, Acetylsalicylic Acid.

## Dabigatran

AEs from the Diversity and the secondary prevention trials are considered.

The Diversity trial was an open-label, randomized, parallel-group, phase 2b/3 non-inferiority trial which evaluated the efficacy and safety of SOC vs. dabigatran for the treatment of VTE in children aged birth to <18 years. The primary composite efficacy endpoint was complete thrombus resolution, absence of VTE-recurrence and absence of VTE-death. In this study, 90 children at a mean age of 11 years and 176 children at a mean age of 11 years were randomized to receive SOC or dabigatran, respectively. Dabigatran was administered as capsules or pellets, and for children <2 years oral solution was available. Results demonstrated that dabigatran was non-inferior to SOC for thrombus resolution and recurrent VTE, showing similar pharmacokinetic/pharmacodynamic relationships to adults. All severe AEs and mild/moderate AEs occurring in more or equal 5% of children as well as all SAEs were reported. Any AEs were reported in 77% of children treated with dabigatran and were similarly distributed across the age groups. The most common AEs were gastrointestinal symptoms in 25%, headache in 10%, and pyrexia in 6%. Of the gastrointestinal symptoms, 32% was vomiting, 27% abdominal pain, and 23% dyspepsia. In 7% of cases, AEs lead to treatment discontinuation. Serious AEs occurred in 13% of children treated with dabigatran. Of these, 14% were bleeding-related, 9% were life-threatening and 77% required hospitalization. There were no deaths of participants taking dabigatran. Fewer children aged from birth to less than 2 years had SAEs (two of 22 children, 9%) than those aged 12 to <18 years (16 of 111 children, 14%). Bleeding events were reported in 38 (22%) of the 176 children randomized to dabigatran. Four children (2%) experienced major bleeding events. One event was a retroperitoneal bleed in a 17-year-old male with cancer which developed 1 week after dabigatran was discontinued. The second event was an episode of hematuria in a 14-year-old child with cancer. The third episode was hematemesis and melena in a 10-year-old child with chronic gastritis. The fourth major bleeding episode was an intracranial hemorrhage in a 1-month-old boy with pneumococcal meningitis. Clinically relevant non-major bleeding (CRNMB) events were reported in 1%, and minor bleeding events in 19% of children. In children aged 0 to <2 years, any bleeding occurred in 6 (27%) of 22 receiving dabigatran. Most of these bleeding events were minor and one was major. Gastrointestinal bleeding (5%), epistaxis (5%), and bruising (3%) were the most common types of bleeding reported (5).

The secondary prevention trial was an open-label, single-arm, prospective cohort phase 3 safety study that investigated the safety of dabigatran for secondary VTE prevention in 203 children at a mean age of 12.8 years after they had completed a 3-month course of anticoagulation for a VTE and had persistent VTE risk factor(s). The primary study end points were VTE recurrence, bleeding events, and mortality. Dabigatran was administered as capsules or pellets. The median dabigatran exposure was 36.3 weeks. Results indicated that dabigatran was safe for secondary VTE prevention in children aged 3 months to <18 years. All severe AEs and mild/moderate AEs occurring in more or equal 5% of children as well as all SAEs were reported. Any AEs were reported in 75% of children, 21% had drug-related AEs. The most common AEs were nasopharyngitis (17%), headache (16%), and abdominal pain (10%). In 6% of children, AEs lead to treatment discontinuation. Twelve percentage of children had SAEs. Of these SAEs, 8% were life-threatening and 92% required hospitalization. The age group birth to 2 years experienced no SAE, while in the age group 12–18 years 12% of children experienced SAE. No deaths occurred in this study. Bleeding events were reported in 20% of the 203 children. Major bleeding events occurred in 3 (2%) children. One event was a bleeding originating from a venous varix in the right leg in a 17-year-old female. Another event was a post-surgical extra pleural hematoma occurring 3 days after a temporary interruption of dabigatran for planned surgery in a 16-year-old. The third bleeding event was an episode of hemoptysis in a 17-year-old male with multiple thrombophilia factors. CRNMB events were reported in 2 (1%), and minor bleeding events in 37 (18%) children. Epistaxis (6%), and bruising (8%) were the most common types of bleeding reported (6).

## Rivaroxaban

AEs from the Einstein Jr. and the Universe trials are considered.

The Einstein Jr. trial was an open-label, randomized, parallel-group, phase 3 trial which evaluated the safety and efficacy of rivaroxaban as compared to SOC for VTE-treatment in children aged birth to <17 years. The primary efficacy outcome was symptomatic recurrent VTE, and the safety outcome was major bleeding or CRNMB. In this study, 165 children and 335 at a mean age of 11.2 years were randomized to receive SOC or rivaroxaban in form of oral solution or tablets. As compared to SOC, rivaroxaban showed a similar low risk of recurrent VTE, but improved thrombus resolution. The study was not powered for non-inferiority outcome. AEs were classified as grade 1–2, grade 3 and 4, with grade 4 being the most severe AE and were evaluated in 329 out of 335 participants receiving rivaroxaban and in 162 of 165 receiving SOC. While all grade 3 or worse AEs were reported, grade 1–2 AEs were only reported if they had occurred in at least 5% of children. Any AEs were reported in 83% of children treated with rivaroxaban. Of these 70% were grade 1–2, 12% grade 3, and 1% grade 4 AEs. Most common AEs were headache in 17%, pyrexia in 11% and gastrointestinal disorders in 33% of cases. Of the gastrointestinal disorders, 32% were vomiting, 17% abdominal pain and 19% nausea. Serious AEs were not reported in this study. One child (1%) in the rivaroxaban treatment group died due to cancer progression, it was not considered treatment related. This was the only reported AE that led to treatment discontinuation. None of the children on rivaroxaban experienced major bleeding events. CRNMB events occurred in 3% of participants. Other relevant bleeding events included one episode (<1%) of pericardial hemorrhage (grade 4) and 2 episodes of procedural hemorrhages (<1%). The most common types of bleeding were epistaxis in 12% of participants, and gingival bleeding in 4%. In addition, 23 (7%) and 4 (1%) episodes of menorrhagia and vaginal bleeding were reported respectively, reflecting a prevalence of 28% calculated from the 97 females aged 12–17-years included in the study (4).

The Universe study was a randomized, multicenter, 2-part, open-label study evaluating rivaroxaban vs. acetylsalicylic acid (ASA) as primary prophylaxis in children who had undergone a Fontan procedure. Primary outcome measures included any thrombotic event as well as major and CRNMB events. In Part A of this study, 12 children at a mean age of 2.5 years received rivaroxaban, in part B 64 children at a mean age of 4 years received rivaroxaban (oral liquid formulation) and 34 children at a mean age of 4 years received ASA. The study was not powered for efficacy, but indicated that children receiving rivaroxaban had less thrombotic events compared to children receiving ASA. Any AEs were reported in 87% of children receiving rivaroxaban. Most common AEs were infections in 63%, respiratory, thoracic and mediastinal disorders in 45% and gastrointestinal disorders in 33% of participants. Four percentage discontinued treatment due to AEs. Serious AE were reported in 32% of children receiving dabigatran. No deaths were recorded. Thirty-six percentage of participants on rivaroxaban had on-treatment bleeding events. One child (2%) had a major bleeding event due to epistaxis, which required a blood transfusion. CRNMB occurred in 7% of children on rivaroxaban. Of these, 40% were gastrointestinal, 40% skin, and 20% were gingival bleeding events. Mild bleeding events occurred in 32% of children on rivaroxaban. Most of these events were epistaxis (29%) and hematomas/skin bleeding (96%). It is important to note, that the post-Fontan surgery participants reflect a single more vulnerable patient population, which is not directly comparable to the standard pediatric VTE population (7, 8).

## Standard of Care (SOC)

AEs from the Diversity, the Einstein Jr. and the Universe trials are considered.

In the Diversity trial, 90 children were randomized to SOC and received either LMWH (44%), VKA (54%) or fondaparinux (1%). Any AEs were reported in 67% of these children, with the lower proportion being in those aged from birth to <2 years (46%) vs. 77% in those aged 12 to <18 years. Gastrointestinal symptoms like vomiting, abdominal pain and dyspepsia were observed in 4%, headache in 4% and pyrexia in 3% of children. In 3% of children, all aged 12 to 18 years, AEs lead to treatment discontinuation. Serious AEs occurred in 20% of children treated with SOC. Of these, 11% were bleeding-related SAEs, 11% were life-threatening SAEs, and 61% required hospitalization. Two (2%) children died, and neither death was considered treatment related. One death was a retroperitoneal bleed in a 15-year-old female with sickle cell crisis 3 weeks after starting SOC treatment for DVT. The other participant had ulcerative colitis, experienced hematochezia and SOC was held, he however then had progression of his CSVT (index event) and a hemorrhagic infarction of which he died 9 days later. Bleeding events were reported in 24% of the 90 children randomized to SOC. Major bleeding events occurred in the same two children who died (see above). Clinically relevant non-major bleeding events were reported in 1%, and minor bleeding events in 23% of children receiving SOC. Epistaxis (7%), bruising (7%) and bleeding from the puncture site (6%) were the most common types of bleeding reported. None of the children aged 0 to <2 years experienced bleeding events (5).

In the Einstein Jr. trial, 162 children were randomized to SOC and received either LMWH (64%) or VKA (34%). Any AEs were reported in 77% of children treated with SOC. Of these 62% were grade 1–2, 14% grade 3, and 1% grade 4 AEs. Gastrointestinal AEs were observed in 28% of participants on SOC, of which 29% were vomiting, 20% were abdominal pain and 16% were nausea. 15% of children had headaches and 8% had pyrexia. There were no AE leading to treatment discontinuation. Serious AEs were not reported in this study. There were no deaths in the SOC group. Overall bleeding events were not reported in this study. Two (1%) of the 165 children on SOC presented with a major bleeding event while on LMWH. One child had an intracranial and another child a pulmonary bleeding event. CRNMB events were observed in 1%. Eleven percentage of participants experienced epistaxis and 4% menorrhagia and vaginal bleeding. The prevalence of menorrhagia and vaginal bleeding was 13% calculated from the 55 females aged 12–17-year-old included in the study (4).

In the Universe study, 34 children were randomized to ASA. Any AEs were reported in 85% of participants. The most common AEs were infections (65%), injury/poisoning/procedural complications (29%) and gastrointestinal disorders (26%). Nine percentage of participants discontinued ASA treatment due to AEs. Serious AEs were reported in 24%. No deaths were reported. Forty-one percentage had on-treatment bleeding events. There were no major bleeding events and CRNMB events occurred in 9% of children on ASA. Of these, 66% occurred in the gastrointestinal tract, and each 33% was located in the skin, subconjunctival tissue and hematoma. One participant could experience more than one site of bleed. Mild bleeding events occurred in 35% of children on ASA. Most of these events were epistaxis (25%) and hematomas/skin bleeding (83%) (7). Of note, the Universe study compares rivaroxaban to ASA – the antiplatelet therapy only. Rivaroxaban is not compared to the SOC such as LMWH or VKA in this study.

## Discussion

In this Mini-review adverse events between the pediatric DOACs, and between the SOCs from the pediatric DOAC trials are summarized and compared. The studies under consideration are the largest studies that have been performed in pediatric populations for treatment of VTEs, and the results are convincing that DOACs are as safe and effective as the respective SOCs. DOACs bring advantages of enteral application and no need for monitoring. The reported cumulative AEs in all four reported trials occurred at similar rates (67–87%). AEs that led to death were rare in all studies, and were not considered treatment related. The low death rate aligns with the low death rate in the adult DOAC studies (9, 10).

The Einstein Jr trial did not investigate serious AEs, subsequently serious AEs are not directly comparable between the different studies. The Universe trial reported a higher number of SAEs in both the rivaroxaban and the ASA groups, as compared to the dabigatran studies. This may reflect the vulnerable congenital heart disease population.

Most AEs within each study were comparable, with no difference observed between the study groups, i.e., not attributable to a specific DOAC or SOC. The most common AEs likely represent common symptoms in the pediatric / adolescent population, independent of the study drugs. This includes gastrointestinal disorders, headaches, nasopharyngitis and pyrexia, which were reported consistently throughout all groups in all trials. As an exception, gastrointestinal AEs were reported distinctly higher for the dabigatran vs. the SOC group in the Diversity study. Pediatric participants treated with dabigatran experienced more dyspepsia, abdominal pain, and vomiting. In adults similar GI AEs have been reported for dabigatran (dyspepsia, GI reflux, dysmotility). Proton pump inhibitors and medication administration with meals have been suggested as symptom interventions (11). Gastrointestinal AEs were however reported at similar rates in the dabigatran secondary prevention trial, the Einstein Jr trial and in the Universe study. Therefore, it is improbable that dabigatran alone is responsible for GI AEs.

Major and CRNMB events were rare overall, and were not related to specific medications. The Universe study reported more bleeding events for both the rivaroxaban and the ASA groups, as compared to the other studies. This is most likely due to the vulnerable patient population, not representing the standard pediatric VTE population. Epistaxis was reported at slightly higher rates in the Einstein trial for both rivaroxaban and for SOC, i.e., not attributable to the one or the other. Menorrhagia and vaginal bleeding were however reported in more participants taking rivaroxaban, than SOC in the Einstein trial. In adults apixaban has been recommended over rivaroxaban in cases with menorrhagia (12). No such recommendations are available presently for pediatric patients.

In Conclusion, dabigatran and rivaroxaban have been proven to be as safe and effective as SOC in the treatment and prophylaxis of pediatric VTE. AEs, major and CRNMB events are overall comparable between the DOACs and SOCs. As DOACs are prescribed more commonly to children, real world data will be generated and experience will be gained in order to assess and reevaluate AEs and bleeding events of DOACs in the pediatric VTE population. Further data and research will confirm or adjust these recommendations for the pediatric population.

## Author Contributions

AB and MA contributed to the design and implementation of the review, to the analysis of the data and to the writing of the manuscript. All authors contributed to the article and approved the submitted version.

## Conflict of Interest

The authors declare that the research was conducted in the absence of any commercial or financial relationships that could be construed as a potential conflict of interest.

## Publisher's Note

All claims expressed in this article are solely those of the authors and do not necessarily represent those of their affiliated organizations, or those of the publisher, the editors and the reviewers. Any product that may be evaluated in this article, or claim that may be made by its manufacturer, is not guaranteed or endorsed by the publisher.
